# Regulatory T-cells and GARP expression are decreased in exercise-associated chikungunya viral arthritis flares

**DOI:** 10.3389/fimmu.2022.1007106

**Published:** 2022-10-07

**Authors:** John E. Dobbs, Sarah R. Tritsch, Liliana Encinales, Andres Cadena, Karol Suchowiecki, Gary Simon, Christopher Mores, Silvana Insignares, Vierys Patricia Villamil Orozco, Mirna Ospino, Lil Avendano Echavez, Carlos Andres Herrera Gomez, Yerlenis Galvis Crespo, Richard Amdur, Alberto David Cabana Jimenez, Carlos Alberto Perez Hernandez, Jennifer Carolina Martinez Zapata, Alfonso Sucerquia Hernandez, Paula Bruges Silvera, Wendy Rosales, Evelyn Mendoza, Estefanie Osorio-Llanes, Jairo Castellar, Dennys Jimenez, Dan M. Cooper, Gary S. Firestein, Karen Martins, Aileen Y. Chang

**Affiliations:** ^1^ School of Medicine and Health Sciences, The George Washington University, Washington, DC, United States; ^2^ Milken Institute School of Public Health, The George Washington University, Washington, DC, United States; ^3^ Allied Research Society, Barranquilla, Colombia; ^4^ Clinica de la Costa, Barranquilla, Colombia; ^5^ Universidad Libre, Barranquilla, Colombia; ^6^ University of Texas Health Science Center San Antonio, TX, United States; ^7^ University of California Irvine, Irvine, CA, United States; ^8^ University of California- San Diego, San Diego, CA, United States; ^9^ Biomedical Advanced Research and Development Authority, Bethesda, MD, United States

**Keywords:** immunology & infectious diseases, viral arthritis, exercise, inflammation, clinical trials, chikungunya arthritis, GARP, chikungunya

## Abstract

**Objective:**

Chikungunya virus (CHIKV) causes persistent arthritis, and our prior study showed that approximately one third of CHIKV arthritis patients had exacerbated arthritis associated with exercise. The underlying mechanism of exercise-associated chikungunya arthritis flare (EACAF) is unknown, and this analysis aimed to examine the regulatory T-cell immune response related to CHIKV arthritis flares.

**Methods:**

In our study, 124 Colombian patients with a history of CHIKV infection four years prior were enrolled and 113 cases with serologically confirmed CHIKV IgG were used in this analysis. Patient information was gathered *via* questionnaires, and blood samples were taken to identify total live peripheral blood mononuclear cells, CD4+ cells, T regulatory cells, and their immune markers. We compared outcomes in CHIKV patients with (n = 38) vs. without (n = 75) EACAF using t-tests to assess means and the Fisher’s exact test, chi-squared to evaluate categorical variables, and Kruskal-Wallis tests in the setting of skewed distributions (SAS 9.3).

**Results:**

33.6% of CHIKV cases reported worsening arthritis with exercise. EACAF patients reported higher global assessments of arthritis disease ranging from 0-100 (71.2 ± 19.7 vs. 59.9 ± 28.0, p=0.03). EACAF patients had lower ratios of T regulatory (Treg)/CD4+ T-cells (1.95 ± 0.73 vs. 2.4 ± 1.29, p = 0.04) and lower percentage of GARP (glycoprotein-A repetitions predominant) expression per Treg (0.13 ± 0.0.33 vs. 0.16 ± 0.24 p= 0.020).

**Conclusion:**

These findings suggest relative decreases in GARP expression may indicate a decreased level of immune suppression. Treg populations in patients with CHIKV arthritis may contribute to arthritis flares during exercise, though current research is conflicting.

## Introduction

Chikungunya virus (CHIKV) infection is associated with severe morbidity, causing fever, rash, myalgias and chronic polyarthritis (1). Both tropical and temperate zones are at risk given transmission by the tropical *Aedes Aegypti* and temperate *Aedes Albopictus* mosquitos (1) resulting in 39% of the global population at-risk for infection (2). The most serious concern of infection with chikungunya is its arthritic symptoms, of which 40% of patients suffer within 3 months of the illness (3). CHIKV’s arthritic symptoms may resemble rheumatoid arthritis (RA) enough to qualify for the diagnostic of the 2010 American College of Rheumatology in some cases (3, 4). While there is an underlying similarity between rheumatoid arthritis and its chikungunya-derived counterpart, there are also differences. For example, while RA has often been associated with reduction in symptoms with exercise (5, 6), chikungunya arthritis patients have worsened symptoms with exercise (7). Our prior cohort study of 123 patients in Barranquilla, Colombia showed that 39% of participants noted that exercise was one of the primary causes of worsened arthritis (7). To date, no study has examined the possible underlying immunologic relationship behind these symptoms. Understanding the underlying pathophysiology and immunology of exercise-associated CHIKV arthritis flares is valuable to identify optimal treatment recommendations.

### Treg functions and arthritis

T regulatory cells (Tregs) play a significant role within the immune system, maintaining the balance between creating peripheral tolerance and eliminating exterior threats and cancers (8). Work by Kulkarni et al. (9) shows there may be an association between proportions of Treg/CD4+ T effector cells and severity of CHIKV arthritis. In addition, the immunologic markers expressed on Tregs, such as GARP (glycoprotein-A repetitions predominant), CTLA-4 (cytotoxic t-lymphocyte-associated-protein 4), Helios and HLA-DR (Human Leukocyte Antigen – DR isotype) can affect Treg immunosuppressive activity (10, 11).

There has also been an association between Tregs and exercise, with consistent exercise leading to increased levels of Tregs, which Dorneles et al. (12) assert leads to lower levels of chronic inflammation. Bartlett et al. (6) found that in a group of 12 individuals with rheumatoid arthritis (RA), ten weeks of interval-walking was able to decrease the disease activity score by 38%, showing the benefits of some types of exercise.

Unlike in RA, there is currently no data documenting the role of T regulatory cells in CHIKV exercise-associated chikungunya arthritis flares (EACAF). While there is evidence that a significant portion of patients suffer from CHIKV EACAF, immunologic data has not been measured. In this analysis, we examined T-cell differences between the patients with and without exercise-associated exacerbation of chikungunya arthritis including CD4+ T effector cells, T regulatory cells and cell markers GARP, CTLA-4, HLA-DR and Helios.

## Methods

### Statement of ethics

This cross-sectional study (IRB no. 121611, Trans no. 28283) was approved by the institutional ethics committees of The George Washington University and the Clinica de La Costa Ltda. All participants received written informed consent and qualified medical personnel were used in the collection of all samples.

### Participants and questionnaires

In this study, 124 Colombian patients with a history of CHIKV infection four years prior were enrolled. Eleven patients were excluded from the analysis due lack of serologic confirmation of CHIKV infection (n=9), lack of blood sample (n=1) or lack of information on exercise effects (n=1). In total, 113 cases were analyzed. Patients were asked to self-identify joint pain associated with chikungunya and to identify if they had other arthritis prior to chikungunya infection. Patients also self-reported when arthritis flares were associated with exercise. Blood samples were collected at the same time that the patients identified having exercise associated chikungunya virus exacerbation. Patients reporting no exercise associated arthritis flares were defined as the CHIKV-controls. Demographic characteristics, disease activity, and arthritis flare information were acquired by physical exam and survey forms.

The Health Assessment Questionnaire (HAQ) was used to assess quality of life, and has often been utilized to assess the level of arthritic disease in individuals (13). Based on of a score of 0-3, an HAQ of 0-1 would indicate mild to moderate disease, 1-2 is moderate severe disease, and a score of 2-3 would indicate severe disease. The PROMIS Mobility score was used as a proxy for level of activity (14). The PROMIS measure helps assess and standardize various evaluations. In this case it will be used to assess overall mobility within this population. The score ranges from 20-80 where 20 indicates the severe issues with mobility, 50 is the average, and 50-80 is within normal limits of mobility. Arthritis disease activity was evaluated *via* the Disease Activity Score-28 (DAS-28) and the patient and physician global assessment of disease severity, which were measured *via* visual analog scale from 0-100. The severity of the flare was assessed *via* an Outcome Measures in Rheumatoid Arthritis Clinical Trials (OMERACT) flare questionnaire by Bartlett et al. (15) adapted for use in CHIKV arthritis to assess pain, fatigue, stiffness, physical function and participation. Each variable was scored from 0-10 which is compounded to 0-50 when aggregated.

### Immunologic evaluation

**Figure 1:**
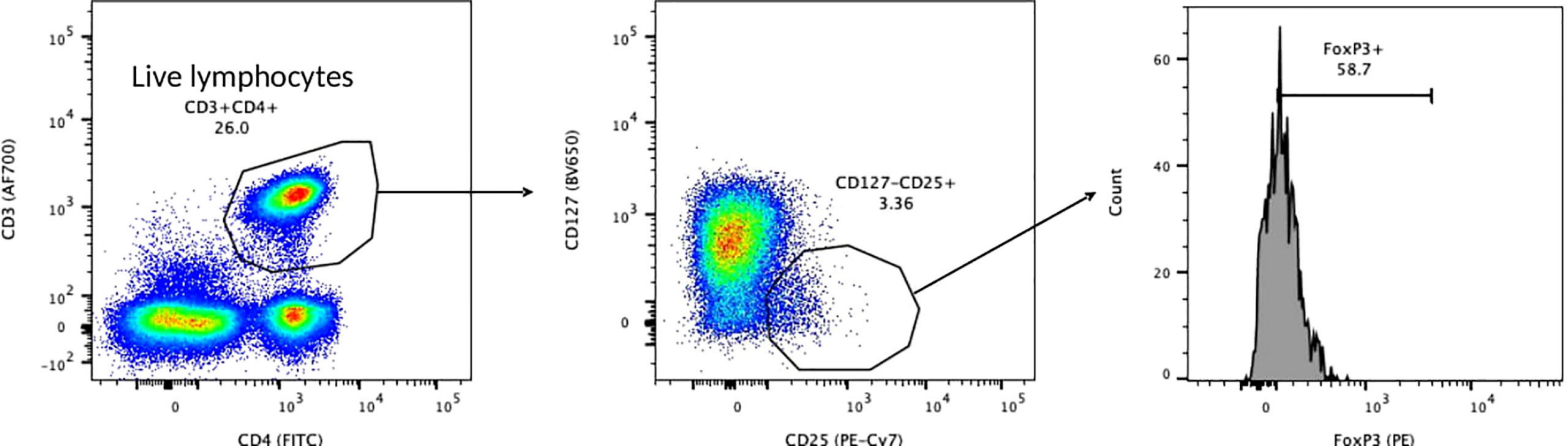
Treg Gating Strategy.

Blood samples were obtained with isolation and cryopreservation of peripheral blood mononuclear cells (PBMCs) with storage at -80 C. Serologic confirmation of chikungunya virus was determined *via* the presence of chikungunya virus IgG antibodies. Slides with biochips that had positive and negative chikungunya cells would be coated with plasma samples. Samples with chikungunya virus IgG would react with the chikungunya cells and cause a fluorescent reaction. Sensitivity and specificity for this test were quite high at 97% and 95% respectively (16). This test was noted to have cross reactivity with IgG for Sindbis and Ross River virus, neither of which have a geographic distribution in Colombia (17, 18). Flow cytometry was performed on thawed PBMCs using a BD Celesta Cell Analyzer. Information on the source, clone, and associated fluorochromes used to identify each marker can be found in **Table 1**. Evaluation of T regulatory (CD3+CD4+CD25hi/int+CD127-FoxP3+), overall CD4+ T cells, (CD3+CD4+) and activation markers CTLA-4, Helios, GARP, and HLA-DR markers were quantified with FlowJo software. The gating strategy for the Tregs can be found in **Figure 1**. The percentage of Treg cells and CD4+ T cells as a proportion of live PBMCs and frequency of CTLA-4, Helios, HLA-DR and GARP were analyzed.

**Table 1:** Flow Cytometry Information.

Marker	Fluorochrome	Source	Clone
**Live/Dead**	Fix Aqua	Thermo Fisher	N/A
**CD3**	Alexa Fluor 700	Biolegend	SK7
**CD4**	FITC	Biolegend	OKT4
**CD25**	PE-Cy7	Biolegend	BC96
**CD127**	Brilliant Violet 650	Biolegend	A019D5
**FoxP3**	PE	Biolegend	206D
**Helios**	APC	Biolegend	22F6
**GARP**	PerCP-Cy5.5	Biolegend	7B11
**HLA-DR**	APC-Cy7	Biolegend	L243
**CTLA-4**	Brilliant Violet 421	Biolegend	BNI3

### Statistical analysis

Comparisons were based on outcomes between CHIKV patients with vs. without arthritis flares due to exercise using a t test, a Fisher’s exact test, chi-squared test, and in the instances of skewed distributions a Kruskal-Wallis test. A Kruskal-Wallis one-way analysis of variance test was used for the immune markers CTLA-4, Helios, GARP, and HLA-DR as a percent of Treg and for comparison of CD4+ cells as a percent of live PBMCs (SAS 9.3, version 9.4).

## Results

Of our 113 patients, 38% self-reported EACAF. EACAF patients are younger by 5 years on average (**Table 2**), with a near equal distribution of sex. The population is mostly of mestizo background. 82% of EACAF patients had at least a high school education compared to 62% of non-EACAF patients. Only 2.63% of EACAF and 8% of non-EACAF patients reported arthritis prior to CHIKV infection. In terms of baseline mobility, PROMIS scores were similar for EACAF and non-EACAF patients at 42.70 and 43.26 respectively with a nonsignificant p value of 0.726. This signifies that both sets of individuals had a mild decrease in mobility relative to the reference population. HAQ scores for both groups also showed mild disease at baseline (0.799 for EACAF and 0.785 for non-EACAF) with no statistical significance. Both patient and physician global assessments for pain (ranging from 0-100) showed statistically significant higher pain assessments for EACAF patients. Statistical significance was not achieved when examining DAS-28 and OMERACT flare scores.

**Table 2:** Results.

	EACAF Patients (N=38)	Non-EACAF patients (N=75)	p Value
Demographics	Mean (SD)	Mean (SD)	
**Age**	45.1 (17.1)	50.4 (16.5)	–
**Percent Female**	83%	84%	–
**Percent with at least Secondary School Education**	82.00%	62.00%	–
**Prior arthritis**	2.63%	8%	–
**Pain & Mobility Scale**			
**HAQ**	0.799 (0.52)	0.785 (0.56)	0.895
**PROMIS Mobility**	42.70 (7.54)	43.26 (9.6)	0.756
**OMERACT**	28.44 (10.77)	25.88 (12.63)	0.289
**DAS-28**	4.06 (1.10)	4.03 (0.95)	0.891
**Patient Global Assessment of Pain**	71.21 (19.72)	59.95 (27.97)	0.028
**Physician Global Assessment of Pain**	57.34 (29.27)	45.64 (28.99)	0.046
**T Cell Analyses**			
**Percent CD4^+^ T cells/Live PMBC**	15.58% (6.86)	16.52% (7.52)	0.543
**Percent Tregs/CD4^+^ T cells**	1.95% (0.73)	2.40% (1.29)	0.044
**Percent GARP+/Treg**	0.13% (0.33)	0.16% (0.24)	0.020
**Percent CTLA-4+/Treg**	19.05% (7.13)	17.77% (7.59)	0.227
**Percent Helios+/Treg**	88.85% (0.75)	88.78% (0.52)	0.952
**Percent HLA-DR_/Treg**	44.60% (12.58)	42.85% (11.99)	0.088

Our analysis of T cells showed a statistically significant difference in the percent of mean Treg/CD4 T cell ratio (1.95 ± 0.73 for EACAF vs. 2.4 ± 1.29 for non-EACAF, p- 0.04). There was also a statistically significant difference in the percent of Tregs expressing GARP (0.13 for EACAF vs. 0.16 for non-EACAF, p- 0.02). Data comparing Helios, HLA-DR, CTLA 4+ or Treg counts did not reach statistical significance.

## Discussion

We found patients with EACAF have a lower proportion of Tregs compared to CD4+ T-cells and lower GARP expression, suggesting a potential role for unregulated cellular responses to mediate inflammation in exercise-associated viral arthritis flares. Chikungunya arthritis is known to be heavily mediated by T-cells. Specifically, CD4+ T cells mediate chronic CHIKV-associated joint swelling (19) and in mouse models, the presence of CD4+ T cells are obligatory for the development of arthritis (19). Kulkarni et al. (9) found that a reduction in regulatory T cells has been associated with the presence of chronic chikungunya arthritis, suggesting that the balance between the development of regulatory T cells and CD4+ T cells may contribute to persistent symptoms. Our research is the first examination of the role of regulatory T cells in exercise-associated flares of chikungunya arthritis and may provide insights for future research directions.

The data from our report show a higher prevalence of females presenting with symptomatic CHIKV arthritis, which concurs with previous findings (7). Based on the data found on PROMIS mobility and HAQ between EACAF and non-EACAF populations, there seems to have been a similar baseline level of disease and mobility. There were individuals with prior arthritis in this study, but it was a small minority for both EACAF and non-EACAF population so this impact should be minimal. We also found that EACAF cases were more likely to have a higher self-reported and physician reported assessment of pain. To the best of our knowledge, this is the first report comparing arthritis disease activity scores for EACAF patients vs. non-EACAF patients.

While there are no studies comparing the effects of exercise on CHIKV arthritis, data from RA patients suggest that contrary to what is found in CHIKV arthritis, exercise seems to reduce disease activity of RA patients (6). The role of Tregs on this effect is still being determined. One randomized control trial (20) of 49 patients with mild RA found exercise reduced the proportion of Tregs relative to CD4+ T cells in females, but did not show a corresponding decrease in disease activity. However, the authors noted that this may be due to the initially low disease activities of patients. Treg activity can mitigate inflammation in CHIKV arthritis, as shown through a murine model (21) and work by Kulkarni et al. (9) demonstrating a relationship between the ratio of Treg/CD4+ T cell and CHIKV arthritis. Interestingly, a metanalysis of 31 studies of RA patients by Morita et al. (22) demonstrated no difference in Treg proportions in RA patients versus healthy controls. However, this metanalysis was complicated by differing gating strategies for defining Tregs among studies where Tregs may have been defined as FOXP3-positive and/or CD25-positive. Further research is needed to determine if there is a causal relationship between exercise, Treg activity and EACAF disease severity.

While the presence of Treg cells can be a marker for self-tolerance, immune markers such as CTLA-4, Helios, HLA-DR and GARP also play important role in immune modulation. A statistically significant decrease in GARP protein expression in Tregs may be indicative of decreased immunosuppression in patients with EACAF (23). GARP protein expression is associated with induced immunosuppression by activating TGF-β1, a cytokine well known for downregulation of the immune system. This decrease in expression of GARP could play a role in both the increase in pain assessment scores and exercise-associated arthritis of EACAF patients. This study also found overall decreased counts in Tregs overall and Tregs expressing HLA-DR, Helios, and CTLA-4 in EACAF patients vs. non-EACAF patients, though none of these changes reached statistical significance. No studies were found in regards to HLA-DR, CTLA-4 and Helios with chikungunya arthritis infection, though lower levels of expression often is related to decreased Treg activity (10, 11, 24). When examined as a proportion of Treg cells, only GARP shows a difference of more than ten percent in EACAF vs. non-EACAF patients. In all of these cases of each marker as a percent of Treg, the distributions were highly skewed, and to address this concern, a Kruskal-Wallis test was used to account for this distribution. In the instance of GARP, there was a statistically significant difference in the population mean despite having overlapping standard deviations of EACAF vs. non-EACAF patients. Further studies are needed with greater power to help elucidate these findings.

Limitations of this study include that differences in weight, height, and BMI, nutritional status were not collected in this study. Types of exercises causing this previously described arthritis exacerbation were also not described. Baseline levels of activity such as household chores were also not documented, though this is mitigated by the collection of PROMIS mobility scores which help establish baseline levels of activity. In this instance, PROMIS scores did not show a statistically significant difference in between populations. Additional limitations include the use of resting cellular analysis. Future studies incorporating blood samples drawn during exercise compared to rest in the same participant and synovial fluid analyses may further define analyses may further define immunologic activity specific to exercise in affected joints. Having more comprehensive data on baseline activity, and height/weight differentiations would also be helpful. It would also be useful to have individuals who were not infected with chikungunya virus to address any baseline joint pains associated with arthritis.

The outcomes of this study suggest a potential role of unregulated CD4+ T-cells in exercise-associated viral arthritis flares. Future research determining the T-cell profile with differing types of exercise and evaluation in a randomized controlled setting with exercise challenge would aid in clarifying this relationship.

## Data availability statement

The raw data supporting the conclusions of this article will be made available by the authors, without undue reservation.

## Ethics statement

The studies involving human participants were reviewed and approved by The George Washington University Ethics Committee; Clinica de La Costa Ltda Ethics Committee IRB no. 121611, Trans no. 28283. The patients/participants provided their written informed consent to participate in this study.

## Author contributions

J D, ACh, ST participated in the design and execution of the study, data analysis, and manuscript preparation. LEn, ACa, KS, GS, CM, SI, VO, MO, LEc, CG, YC, AJ, CH, JZ, AH, PS, WR, EM, EO-L, JC, DJ, DC, GF, KM contributed in patient interviewing, sample preparation, data interpretation, and manuscript editing. RA contributed in the data analysis of the correlation of the clinical manifestations of the trial with the cellular data retrieved within the immunologic evaluation. All authors contributed to the article and approved the submitted version.

## Funding

Research reported in this publication was supported by the National Institute Of Arthritis And Musculoskeletal And Skin Diseases of the National Institutes of Health under Award Number K23AR076505. The content is solely the responsibility of the authors and does not necessarily represent the official views of the National Institutes of Health.

## Conflict of interest

Authors LE, SI, VO, MO, and EM, were/are employed by the Allied Research Society.

The remaining authors declare that the research was conducted in the absence of any commercial or financial relationships that could be construed as a potential conflict of interest.

## Publisher’s note

All claims expressed in this article are solely those of the authors and do not necessarily represent those of their affiliated organizations, or those of the publisher, the editors and the reviewers. Any product that may be evaluated in this article, or claim that may be made by its manufacturer, is not guaranteed or endorsed by the publisher.
